# Atomically Precise Control of Topological State Hybridization in Conjugated Polymers

**DOI:** 10.1021/acsnano.4c10357

**Published:** 2024-10-15

**Authors:** Alejandro Jiménez-Martín, Zdenka Sosnová, Diego Soler, Benjamin Mallada, Héctor González-Herrero, Shayan Edalatmanesh, Nazario Martín, David Écija, Pavel Jelínek, Bruno de la Torre

**Affiliations:** † Regional Centre of Advanced Technologies and Materials, Czech Advanced Technology and Research Institute (CATRIN), Palacký University, 78371 Olomouc, Czech Republic; ‡ Institute of Physics of the Czech Academy of Sciences, 16200 Prague, Czech Republic; § Faculty of Nuclear Sciences and Physical Engineering, Czech Technical University, 11519 Prague, Czech Republic; ∥ Departamento de Física de la Materia Condensada, Universidad Autónoma, E-28049 Madrid, Spain; ⊥ Condensed Matter Physics Center (IFIMAC), Universidad Autónoma, E-28049 Madrid, Spain; # Departamento de Química Orgánica, Facultad de Ciencias Químicas, Universidad Complutense, 28040 Madrid, Spain; ∇ IMDEA Nanoscience, Campus Universitario de Cantoblanco, 28049 Madrid, Spain; ○ Nanomaterials and Nanotechnology Research Center (CINN), CSIC-UNIOVI-PA, 33940 El Entrego, Spain

**Keywords:** topological quantum phase transition, π-conjugated polymers, atomic manipulation, scanning tunneling microscopy, noncontact atomic force microscopy

## Abstract

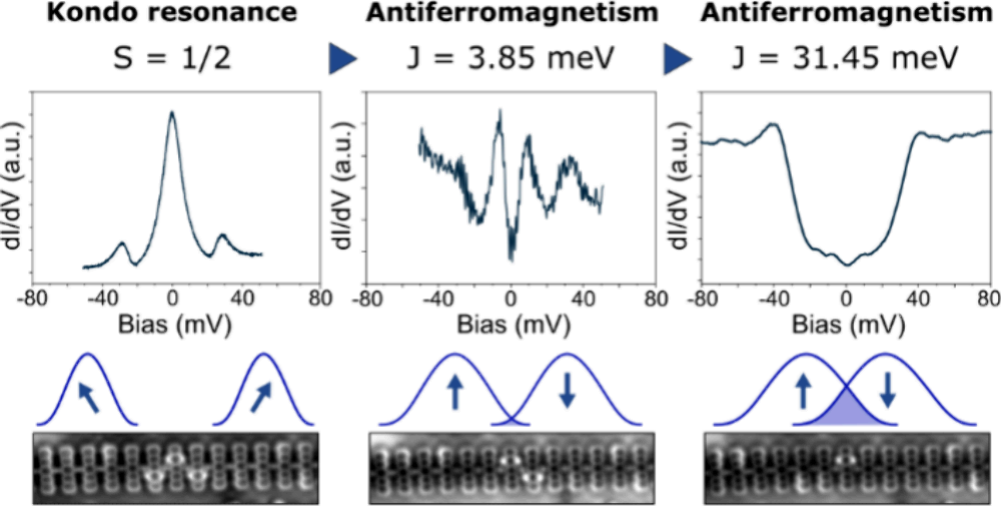

Realization of topological quantum states in carbon nanostructures has recently emerged as a promising platform for hosting highly coherent and controllable quantum dot spin qubits. However, their adjustable manipulation remains elusive. Here, we report the atomically accurate control of the hybridization level of topologically protected quantum edge states emerging from topological interfaces in bottom-up-fabricated π-conjugated polymers. Our investigation employed a combination of low-temperature scanning tunneling microscopy and spectroscopy, along with high-resolution atomic force microscopy, to effectively modify the hybridization level of neighboring edge states by the selective dehydrogenation reaction of molecular units in a pentacene-based polymer and demonstrate their reversible character. Density functional theory, tight binding, and complete active space calculations for the Hubbard model were employed to support our findings, revealing that the extent of orbital overlap between the topological edge states can be finely tuned based on the geometry and electronic bandgap of the interconnecting region. These results demonstrate the utility of topological edge states as components for designing complex quantum arrangements for advanced electronic devices.

## Introduction

The discovery of topological quantum phases within confined carbon-based nanostructures, particularly graphene nanoribbons (GNRs),^1−4^ has sparked considerable interest due to recent experimental and theoretical advancements.^3,4^ These advancements have positioned carbon nanostructures as promising candidates for fundamental components in both classical and quantum information processing technologies.^5−8^

Carbon-based nanostructures possess properties that make them highly desirable for such applications. First, they exhibit minimal sources of decoherence, including spin–orbit coupling and hyperfine interaction.^1^ Second, the bandwidth of the topological electronic band in these nanostructures can be precisely adjusted, particularly near the energy scale of proximity-induced spin–orbit coupling. Furthermore, topological edge states at GNRs emerge as particularly promising elements for incorporation into quantum information devices.^5,7^

Over the past five years, topological bound states in carbon nanostructures have been deliberately engineered through the bottom-up on-surface synthesis strategy.^9−12^ This method has facilitated precise control over their structural and electronic properties, leading to the emergence of topological states within the band structure. The concept of bulk-edge correspondence ensures the existence of boundary states in topological systems, such as GNRs, which are protected by the energy gap and the nontrivial topology of the bulk states.^2,13,14^ As a result, interfaces formed between segments of carbon-based nanostructures with differing topological invariants can give rise to topological edge states.^15−17^ These states experimentally manifest near the midgap region of the energy spectrum and exhibit the potential for hosting localized spins under specific conditions. This method has enabled the fabrication of qubits, quantum spin chains, and one-dimensional band structures.^16,18−22^ The required presence of boundaries, including atomically defined junctions and finite terminations, necessitates in general the use of approaches involving the covalent fusion of different molecular precursors or defects.^19,22−26^ However, the low-energy spectra of such quantum dots are ultimately determined by electron hopping amplitudes and subsequent atomic rearrangements, which cannot be reversibly altered in such covalent nanostructures. Therefore, an additional element that would significantly enhance the functionalities and broaden the range of potential applications for these carbon-based materials would be the selectivity and switchability of their properties.

Here, we report the selective and reversible manipulation of the hybridization level of topological edge states that emerge at the interfaces between segments of one-dimensional pentacene polymers with different topological invariants. We have utilized local probe-induced single-molecule chemistry^27−29^ to manipulate neighboring topological heterostructures connected by well-defined polymer interfaces. The resulting custom-made heterojunctions are separated by an atomically precise adjustable distance, thereby ensuring the creation of topological edge states with tunable hybridization across defined polymer segments with a predetermined electron wave function overlapping amplitude. Utilizing a combination of low-temperature scanning tunneling microscopy (STM) and spectroscopy (STS) with noncontact atomic force microscopy (AFM),^30,31^ we conducted a comprehensive characterization of several configurations fabricated under ultrahigh vacuum conditions (UHV). Our empirical observations, supported by density functional theory and tight binding calculations, elucidate that the edge states are spin polarized. Left and right edge spins polarized to opposite directions forming a topological spin qubit with capacity to reversibly tune the magnitude of exchange between topological edge states. These results emphasize the effectiveness of topological edge states as essential building blocks for crafting and deploying customized quantum dots.^32^ Additionally, they provide valuable insights into investigating topological phenomena within a many-body physics framework.

## Results

### Setup and Model

**Figure 1:**
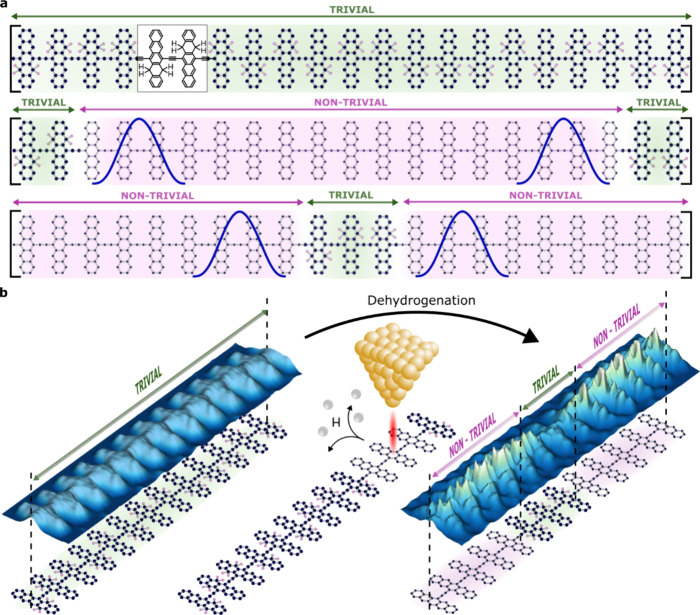
Model and setup of the pentacene polymers. (a) Schematic representation of different configurations for the hydrogenated pentacene polymer and two different topological heterostructures after the dehydrogenation process. (b) Tip-induced dehydrogenation setup for the formation of the nontrivial region inside the pentacene polymer from the hydrogenated trivial phase. Note for chemical representation: Atoms in pink represent hydrogen, while atoms in black represent carbon.

Figure 1a depicts a schematic representation of the assembly process for two distinct topological heterostructures featuring hybridized edge states. In the initial structure, two edge states within a single topological segment are sufficiently proximate to facilitate wave function overlap. In the alternative configuration, two distinct topological segments are partitioned by a diminished semiconducting tunnel barrier, allowing for the hybridization of the edge states.

In both configurations, we leverage the atomic scale tunability inherent in the topological phase transitions exhibited by pentacene polymers. The generation of the topological states in pentacene polymers starts from the readily described on-surface synthesis of pristine polymers on Au(111).^33^ These pristine polymers undergo a length-dependent topological quantum phase transition induced by the pseudo-Jahn–Teller effect. The change of topological state of pentacene polymers is accompanied by a change in their π-conjugation (i.e., their resonance form), from ethynylene-aromatic (topologically trivial) to cumulene-quinoid (topologically nontrivial). This change in the character of the π-conjugation is followed by the emergence of topologically protected edge states.^34^

In this study, we utilize a two-step process previously introduced^34^ that combines hydrogen chemistry with local probe chemistry to manipulate the topological phase in targeted polymer segments. This method entails subjecting pentacene polymers to atomic hydrogen within a UHV chamber. It results in the selective adsorption of two hydrogen atoms at either the 7,12 or 5,14 positions of the pentacene core, thereby forming double-hydrogenated pentacene polymers. The high specificity of the hydrogen adsorption process is driven by the creation of a different π-resonance form with two Clar’s sextets, minimizing the total energy of the hydrogenated product. Consequently, this process substantially increases the electronic bandgap of the polymer (from 0.35 to approximately 1.8 eV) and facilitates the controlled generation of extended pristine pentacene polymers with trivial topology.^34^ The extra hydrogen pair can be systematically removed by positioning the STM tip directly above one of them,^35,36^ as depicted in Figure 1b, and recording a distance versus voltage curve (see Figure S1). This procedure involves gradually increasing the voltage from −1.5 to −3.0 V while maintaining a constant current of 10 pA (see Methods in Supporting Information). Through the iterative implementation of this technique, specific regions of pristine pentacene polymers surrounded by hydrogenated segments can be selectively engineered, thereby modifying their topological phase in accordance with their length and exposing the edge states to different heterojunctions.

### Magnetic Nature of the Zero-Bias Resonance

**Figure 2:**
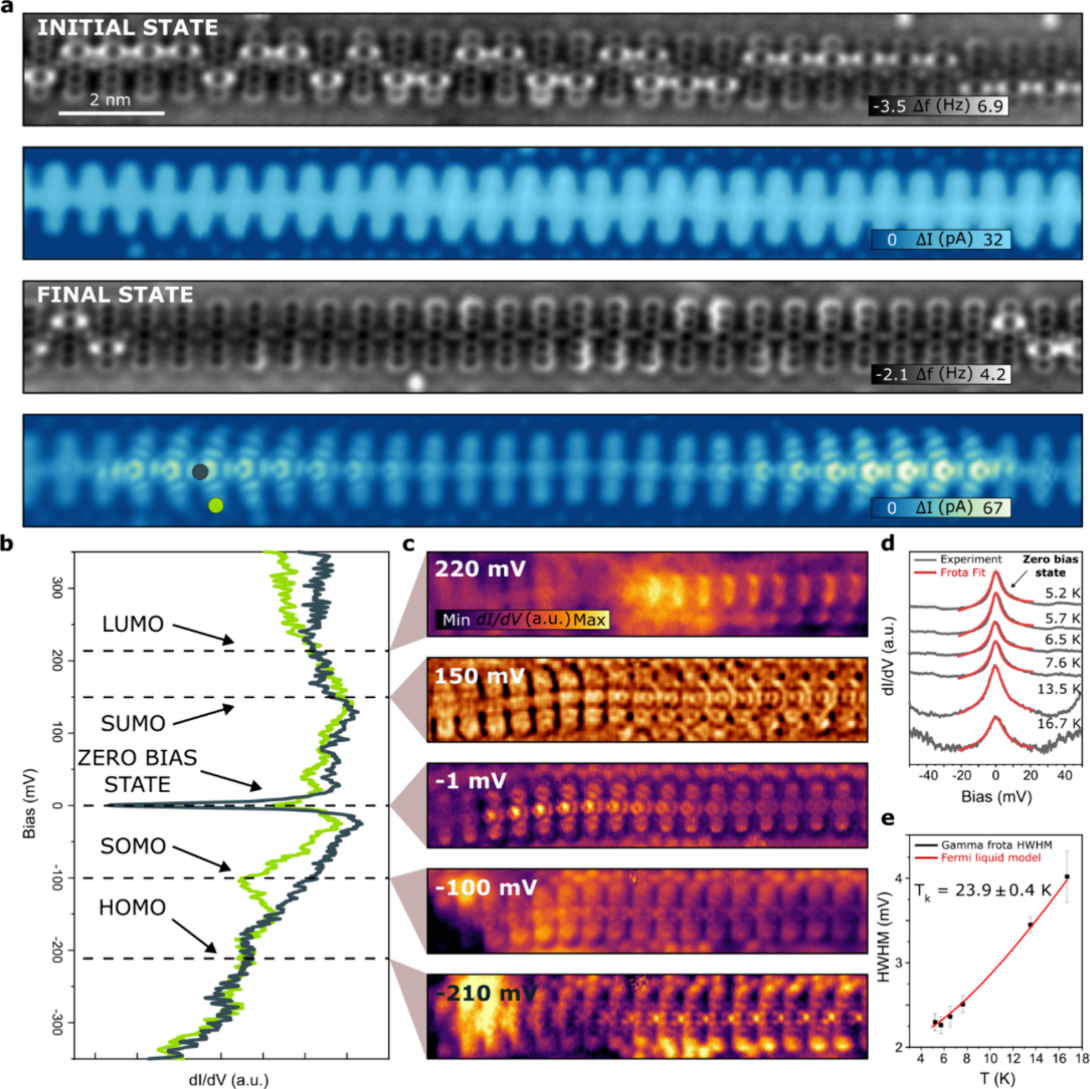
Characterization of the zero-bias resonance. (a) Initial and final states of the quantum phase transition during the dehydrogenation process showing the appearance of the edge state (*V*_b_ = 5 mV; *I*_t_ = 10 pA). (b) d*I/*d*V* spectra of the topological edge state. (c) Differential conductance maps of the LUMO (220 mV), SUMO (150 mV), ZBR (−1 mV), SOMO (−100 mV), and HOMO (−210 mV) at the end of a nontrivial region of the pentacene polymer. (d) Evolution of the Kondo state with the temperature (gray) fitted by Frota function (red). (e) Half width at the half-maximum (HWHM) values of the Kondo state as a function of the temperature. Kondo temperature results in *T*_K_ = 23.9 ± 0.4 K.

Initially, we investigate in detail the electronic properties of the edge states, extending beyond the previous mere observations of the emergence of topological states in pentacene polymers.^33,34^Figure 2a illustrates detailed images captured by AFM and STM of a pentacene polymer segment composed of more than 30 hydrogenated units (initial state), where the dehydrogenation process was executed in 24 pentacene units (final state) utilizing a CO-functionalized probe. At a low-bias voltage of 5 mV, the STM image showcases enhanced contrast at the edges of the pristine segment due to the presence of zero-bias states. Figure 2b depicts the differential conductance spectrum (d*I/*d*V*) collected at specific positions near the edge state, as indicated in Figure 2a. Besides the highest occupied and lowest unoccupied molecular orbitals (HOMO and LUMO) of the pentacene polymer at −0.21 and 0.22 V, a zero-bias resonance (ZBR) is clearly resolved aligning with previous reports.^33,34^ The identification of a ZBR in spectra obtained at the edges which widens unusually rapidly with temperature (Figure 2d), indicates evidence of a net spin *S* = 1/2 and the emergence of a Kondo resonance due to the screening of the local magnetic moment.^37,38^ To prove the Kondo nature of a zero-bias peak, it needs to be shown that it follows the appropriate behavior as a function of a particular parameter that distinguishes it from all other possible ZBRs. The most conventional approach involves varying the temperature and examining its effect on the peak width in the absence of a magnetic field. In Figure 2e, the half width at half-maximum (HWHM) of the temperature-dependent spectra, derived from fits to a Frota function, is examined using the Fermi liquid model, yielding a Kondo temperature of 23.9 ± 0.4 K. The Anderson model for magnetic states on metal substrates^38,39^ predicts two resonances in the density of states: one below the Fermi level for the singly occupied molecular orbital (SOMO) and another above the Fermi level for the singly unoccupied molecular orbital (SUMO) separated from the occupied state by the charging energy U.

The differential conductance spectrum in Figure 2b does not clearly resolve specific electronic resonances that can be attributed to the singly occupied states. This ambiguity is likely due to the hybridization of the SUMO and SOMO states with the HOMO and LUMO, owing to the low bandgap of the polymer and the resultant wave function overlap. To further investigate this, we acquired a set of d*I/*d*V* maps at various voltages within the polymer bandgap (Figures 2c and S2). These maps reveal a significant density of states along the polymer center, similar to the Kondo map, emerging at energies −100 and 150 meV within the polymer bandgap. The comparable local density of state (LDOS) distribution at these energy levels in d*I/*d*V* maps supports their shared origin from the SOMO/SUMO, separated by a small Coulomb gap of 0.25 eV. Interestingly, the non-negligible hybridization of the radical states with the frontier molecular orbitals results in a notably extended distribution into adjacent unit cells. The consistent spin distribution of the Kondo map obtained at 5 mV and of the SOMO/SUMO provides supplementary evidence for the origin of the zero-bias state. The intensity of the Kondo resonance dominates over the first few lattice sites within the last monomer unit but fades as moving toward the polymer bulk (Figure S3), consistent with experimental d*I/*d*V* mapping at −1 mV (Figure 2c). Specifically, we found that the Kondo state extends up to 8 units, with a maximum intensity localized at the third one (from the edge) that continuously decreases into the polymer bulk, highlighting sufficient stability to prevent spin quenching upon absorption on the metal surface.^40^ This observation indicates the potential to adjust the extension of the polymer to control nontrivial segments, where both edge states hybridize because of sufficient wave function overlap leading to an extended metallic state.^16^

### Fabrication of Hybridized States in Pristine Polymer

**Figure 3:**
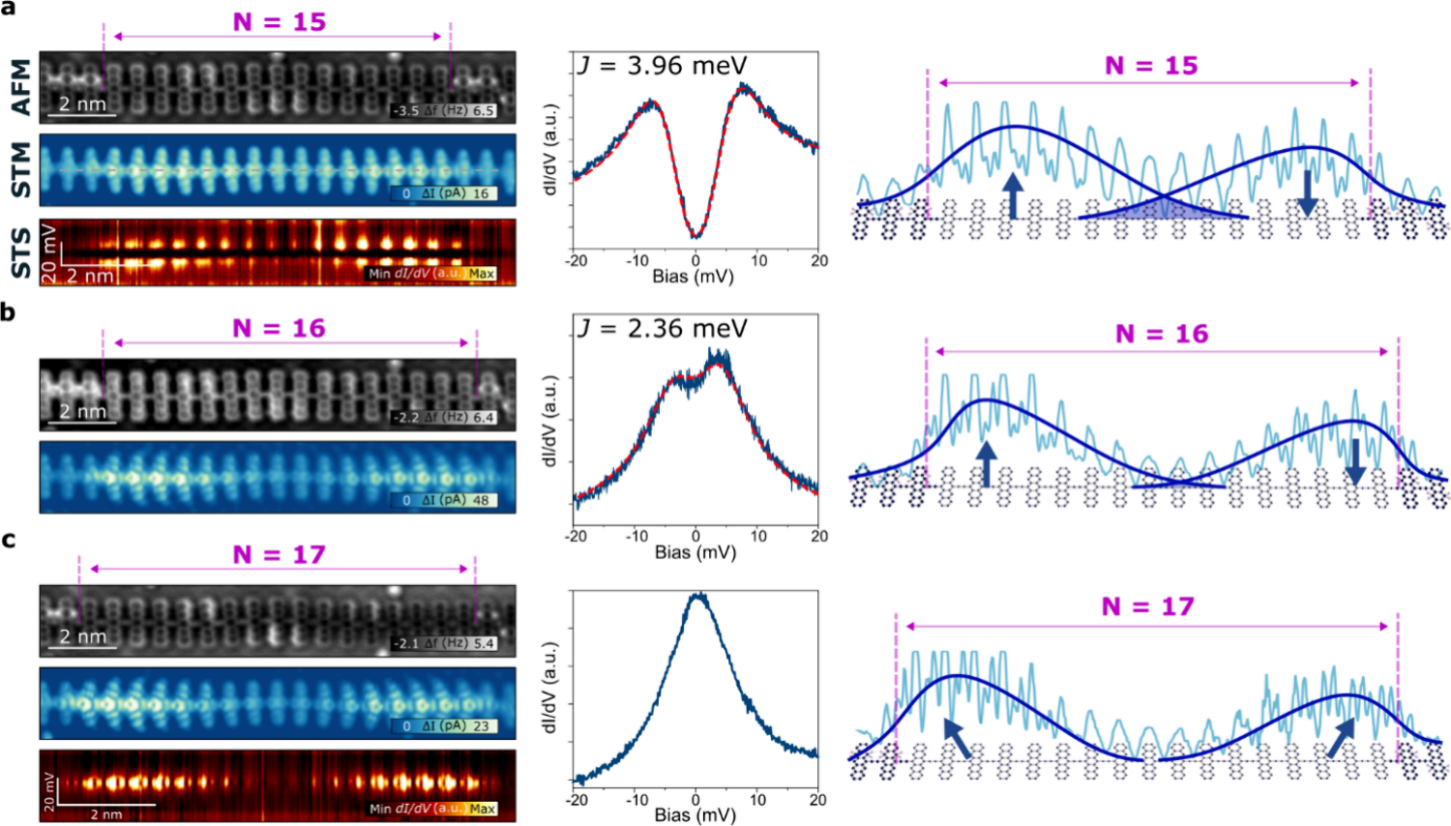
Topological edge states in a pristine pentacene polymer. AFM, STM, line STS, punctual STS, and a model with superimposed current profile in a segment of (a) *N* = 15, (b) *N* = 16, and (c) *N* = 17 dehydrogenated pentacene units. Characterization shows a transition from a magnetic coupling exchange of two topological edge states to an isolated Kondo-type resonance inside a pristine polymer. Antiferromagnetic exchange is *J* = 3.96 meV and *J* = 2.36 meV for *N* = 15 and *N* = 16 cases, respectively (*V*_b_ = 5 mV; *I*_t_ = 10 pA).

With the aim of exploring this possibility, several dehydrogenated pentacene polymer segments ranging from 0 to 24 were fabricated, each manifesting distinct electronic properties (see Figure S4). Analysis via low-bias STM imaging revealed that up to 14 unit segments exhibited faint extended contrast devoid of the zero-energy state. This is a consequence of its trivial semiconducting nature.^34^ Contrary, the 15 and 16 unit segments showcased intensified contrast indicative of the ZBR extending longitudinally across the entire segment, albeit with diminishing intensity within the bulk of the polymer, indicating a topological phase transition. We should note that this observation marks the onset of the topological transition at 15 units in extended hydrogenated pentacene (Figure 3a), differently to the previous reported transition at some length above 25 units. In the former case, it is important to highlight that higher molecular coverage was employed to form larger polymeric structures. As a result, the polymers have limited space to diffuse, leading to a parallel arrangement on the surface, which affects their alignment with the substrate, unlike in previous studies. Recent literature indicates that mechanical strain has the potential to influence the topological transition, thereby affecting the tuning of the bandgap in pentacene polymers.^41^ This suggests the possibility of adjusting the precise point at which the topological transition occurs by the atomic register with the surface. Additionally, the use of longer molecular chains may influence the topological transition driven by weak electron–phonon coupling.^34^

In contrast to the previously discussed scenario concerning larger polymer segments, the low-energy spectra acquired at the polymer terminus exhibited two symmetric peaks at ±7 and ±4 meV for the 15 and 16 units, respectively, as shown in Figure 3a,b. This observation correlates well with an *S* = 0 singlet ground state,^42−45^ characterized by the preferred antiferromagnetic (afm) alignment of electrons in chemical bonds. The validation of the exchange coupling energies was achieved through spectral fitting employing a perturbative methodology for two coupled *S* = 1/2 systems utilizing the code developed by Ternes, as elucidated in pertinent literature.^46^ Then, the hybridization is characterized by an effective exchange parameter *J* of 3.96 and 2.36 meV for the 15 and 16 units case, respectively. The observation of the magnetic exchange coupling in different pentacene polymers can differ maybe due to subtle mechanical strain or conformational changes^41^ (see Table 1 in SI).

Herein, it is important to highlight that magnetic coupling in a small nanographene (Clar’s Goblet), triangulene dimers, and GNR junctions on metal surfaces has been recently reported, revealing how their antiferromagnetic ground state survives in contact with a metallic surface.^40^ Interestingly, the afm exchange coupling for the 15 and 16 units pentacene polymer appears despite the presence of the cumulene-like bridging unit with sp-bonding character. This indicates that the antiferromagnetic coupling is not limited to sp^2^-hybridization.^47^

The configuration of the hybridized state is clarified through line spectroscopy performed along the polymer segment, indicating delocalization throughout the polymer extension (Figure 3a). The edge state coupling favored a closed-shell ground state with a doubly occupied HOMO due to the presence of a large hybridization energy capable of overcoming the Coulomb repulsion between electrons.^48^ It is noteworthy that our experimental line spectroscopy conducted on the 15 unit pentacene polymer confirms the comprehensive delocalization of the diradical state along the entire segment extension, spanning approximately 10 nm (Figure 3a). In this regard, recent research has suggested that extended topological radical pairs could serve as ultrahigh conductance molecular wires. Ultrahigh conductance is attributed to the emergence of reversed conductance decay facilitated by the coupling of energetically low-lying topological edge states which typically decreases with increasing length,^49−51^ thus far been limited to molecular systems on the order of a few nanometers.

Focusing on the exchange coupling, it scales inversely with the spatial separation of the edge states. That is, radical pairs separated by fewer unit cells (shorter polymers) show larger *J* values due to the greater overlap of the states’ wave functions and the associated spin density (Figure 3a,b). The afm coupling between two ends becomes negligible (paramagnetic-like behaviors) as the length of the pentacene polymer reaches 17 unit cells, wherein Kondo resonance peaks can be observed at both termini (Figure 3c). This length-dependent spin interaction aligns with the observed trend in diradical peripentacene polymers on Au(111), wherein there is a consistent decrease in the afm coupling strength between terminal spins as the length of the diradical polymer increases.^52^ In comparison, for the peripentacene scenario, its larger bandgap (≈0.8 eV) leads to increased localization of radical states at both termini, thereby reducing the level of hybridization. Consequently, hybridization in the peripentacene is only evident for the dimer, while the trimer already exhibits separate Kondo signatures at both termini.

### Interpolymer Coupling in Topological Heterostructures

**Figure 4:**
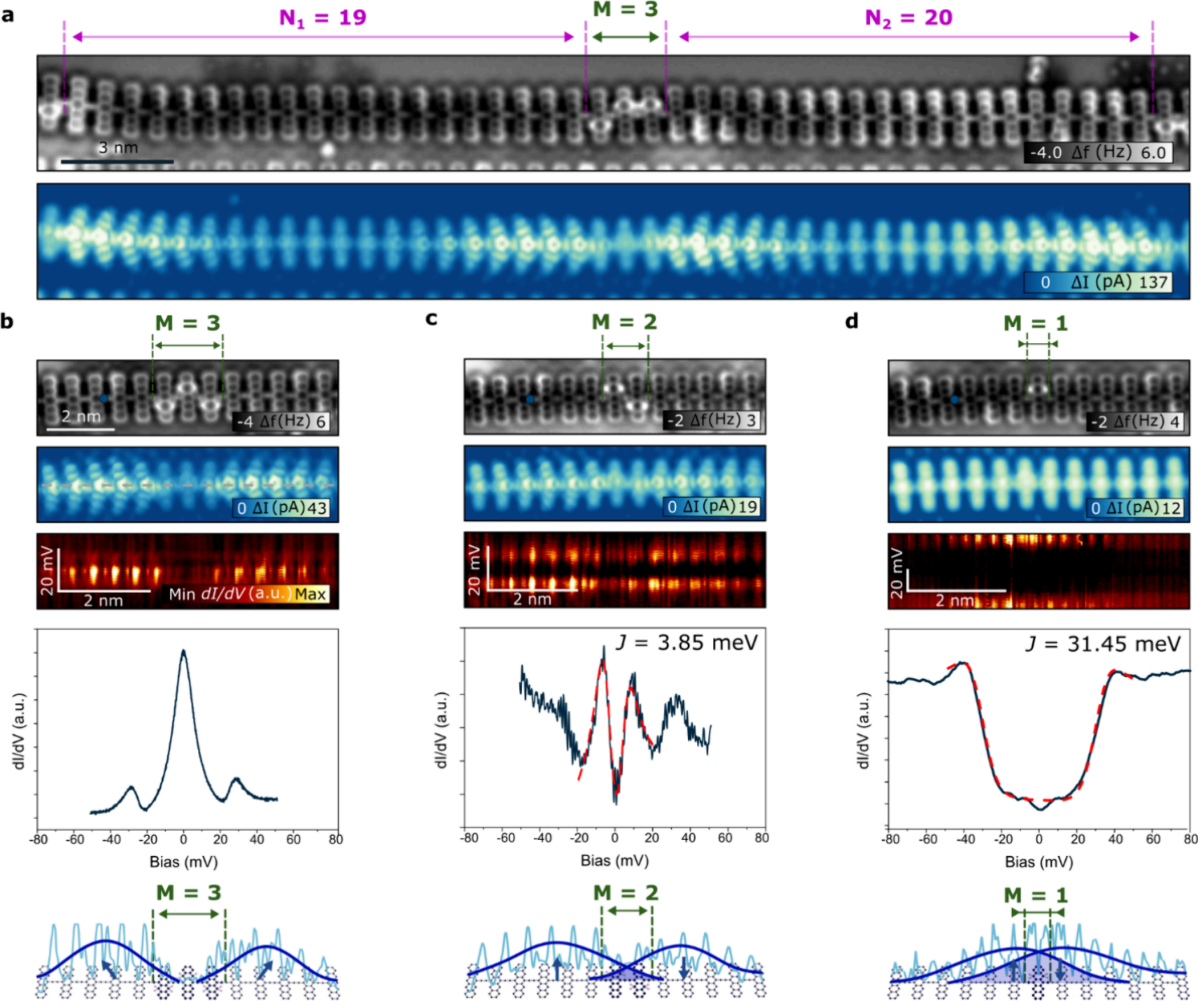
Interpolymer edge state coupling in topological heterostructures. (a) Large-scale AFM (top) and STM (bottom) images of a topological heterostructure inside the pentacene polymer. (b–d) Topological heterostructures with a semiconductor barrier of *M* = 3, *M* = 2, and *M* = 1 hydrogenated units, respectively, and their AFM, STM, line STS, punctual STS, and representation with the current profile of each case. Profile in the model of the *M* = 1 case corresponds to the line d*I/*d*V* image taken at approximately 40 mV. Antiferromagnetic exchange is *J* = 3.85 meV and *J* = 31.45 meV for *M* = 2 and *M* = 1, respectively. Polymer in the panel (a) does not correspond to the polymer in the panel (b) (*V*_b_ = 5 mV; *I*_t_ = 10 pA).

Our next objective is to create heterostructures consisting of topological segments separated by trivial polymer portions, as represented in Figure 4a. Specifically, we seek to investigate the potential hybridization of closely positioned topological states through a semiconductive tunnel barrier to probe a more realistic scenario for potential quantum applications. To achieve this, we followed the two dehydrogenation processes outlined previously. Beginning with an extended hydrogenated pentacene polymer comprising approximately 50 units, we initially produced two adjacent topological segments. Each contains more than 17 units to prevent the hybridization of edge states within a single pentacene segment, that is, featuring only the topological states associated with the *S* = 1/2 at both termini. The precise formation of the topological heterostructure is illustrated in Figure S5. Notably, these topological segments are separated by numerous hydrogenated units, which, as previously described, possess a wider band gap (approximately 1.8 eV) compared to the smaller band gap of the topological pentacene polymer. This arrangement serves as the semiconducting barrier between the two nontrivial segments. Subsequently, we meticulously examine the coupling of neighboring edge states by sequentially reducing the barrier one unit at a time through controlled hydrogen pairs removal. It should be emphasized that more intricate structures involving the examination of the coupling between adjacent *S* = 0 afm spin states (namely, for nontrivial segments comprising 15 or 16 dehydrogenated units) may warrant further in-depth investigation as the coupling between multiple nanocavities in close vicinity leads to the hybridization of their modes providing a route to fabricate zero-mode superlattices.^16^

In scenarios where the barrier consists of three or more hydrogenated pentacene units, low-bias spectroscopy obtained at the nontrivial segment termini near the barrier reveals only the ZBR associated with Kondo screening (Figure 4b). Additionally, two peaks at ±35 mV associated with the frustrated rotational vibration of the CO-tip are resolved.^53^ The corresponding low-bias (5 mV) STM image displays conductance enhancements that gradually decrease toward the bulk of the nontrivial segment and rapidly decline toward the barrier, due to the significantly different bandgap. This observation is corroborated by the line spectroscopy acquired along the marked line in Figure 4b. By reducing the barrier to only two hydrogenated units, the situation undergoes a drastic change. Now, the low-bias spectra at both terminal states near the barrier exhibit two peaks at symmetric energies characteristic of afm spin coupling (Figure 4c), akin to the case of pristine pentacene polymer with 15 and 16 units. The measured exchange coupling in this instance is 3.85 meV, which closely resembles that obtained for 15 pentacene units, albeit over a much shorter distance between the coupled edge states. This value is consistent with others values of *J* obtained in heterostructures of different pentacene polymers (see Table 2 in SI). This comparison can be intuitively understood as two topological states connected through either a long narrow-bandgap barrier (as in the case of 15 pentacene units) or a short wide-bandgap barrier (as in the case of two hydrogenated units), demonstrating the ability to selectively tune the hybridization of topologically protected states using different mechanisms within a single molecular polymer. A non-negligible state density can be observed penetrating the barrier in the line spectroscopy, as presented in Figure 4c.

Continuing our investigation, we proceeded to reduce the barrier to only a single H-pentacene unit positioned between the two edge states. Although the STM image taken near the Fermi level did not reveal any discernible edge states, the spectra acquired over a narrow energy range displayed notable conductance enhancements, with symmetric step-like features appearing at approximately ±40 meV. While these symmetric steps have traditionally been associated with spin-flip excitations of the *S* = 1 triplet state, they could also signify *S* = 0 antiferromagnetic coupling with a sizable *J*.^46^ In our analysis of the line spectroscopy, we observed a significant amplitude of the hybridized state at the barrier, which exhibited a rapid decline toward the nontrivial segment bulk. Consistent with our previous discussions, we found that the *J* energy increased inversely with the distance between the edge states with an obtained value of 31.45 meV. This observation underscores the nuanced relationship between hybridization and distance in pentacene polymer systems, highlighting the intricate interplay between molecular structure and electronic properties, and demonstrating their potential for testing complex topological heterostructures.

### Theoretical Calculations

To gain deeper insight into the variation of the exchange coupling *J* between neighbor ZBRs mediated by the semiconducting barrier, we employ the many-body Complete Active Space (CAS) method, resolving the Hubbard model.

First, we employed the Hückel model with a parabolic distance-dependent hopping to describe the electronic structure of the π-system of the pentacene polymers (see SI). We set the default distance between carbon atoms to be *d* = 1.4 eV in the model. To induce the nontrivial phase with ZBR in pentacene segments, we increase the distance *d*_*p*_ between pentacene units, as detailed in the SI (see Figures S6 and S7). Likewise, we reduce the distance of the bonds on the bridges between anthracene units to position the segment in the topologically trivial phase and mimic the large band gap of the hydrogenated pentacene polymer (1.5 eV).

To study the experimentally observed dependency of the antiferromagnetic exchange coupling *J* between the ZBRs on the number of hydrogenated units in the barrier, we solved the Hubbard model (for details, see SI) using the CAS method. We performed the exact diagonalization in a reduced active space of six molecular orbitals obtained from the one-electron Hückel model, as shown in Figure S8. From the many-body CAS calculation, we extracted the difference between the singlet ground state and the first excited triplet state for different numbers of anthracene units. This predicts spin excitations of 40 and 5 meV, respectively, for the chains with one and two anthracene units, in good agreement with the experimental exchange coupling, as well as the rapid transition to the degenerate regime, where the exchange coupling *J* is effectively zero (see Figure S9).

### Reversible Hydrogenation

**Figure 5:**
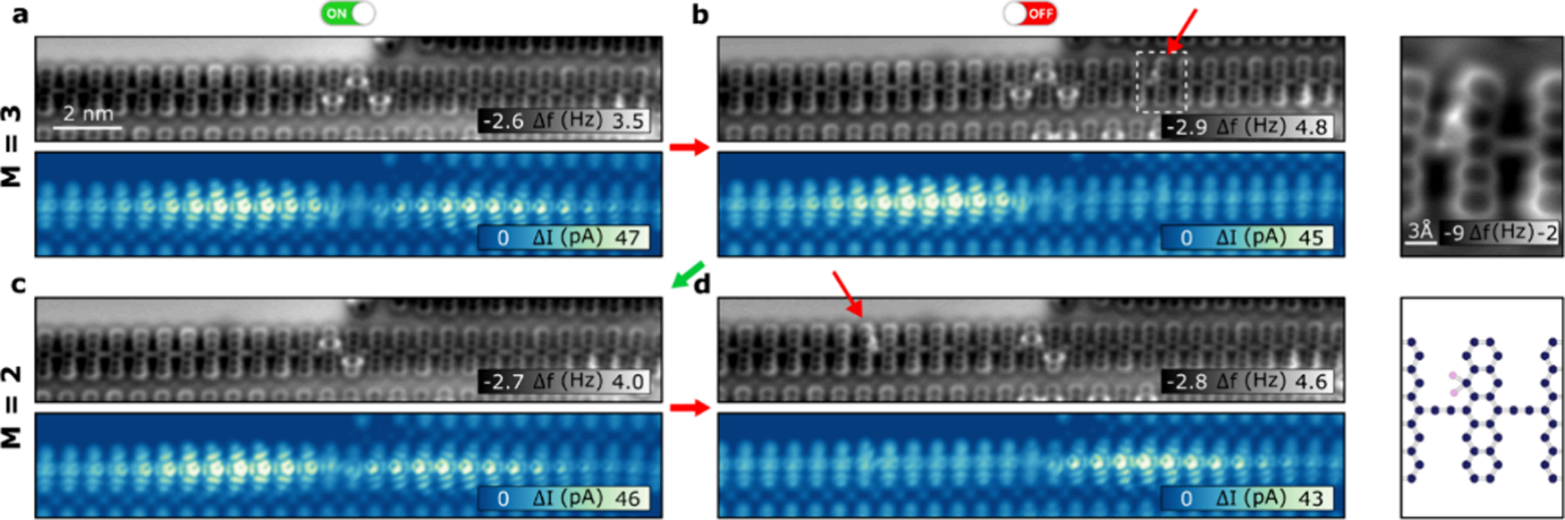
Reversible hydrogenation. (a) AFM and STM images of a topological heterostructure with an interface of *M* = 3 hydrogenated units with both states ON. (b) Addition of a single H atom to the structure producing an OFF state. (c) AFM and STM images after dehydrogenation of the extra single H with and interface of *M* = 2 with the states ON. (d) Sudden hydrogenation allows switching to the OFF state in a reversible way. Inset image shows an AFM detail of the single hydrogenated unit (*V*_b_ = 5 mV; *I*_t_ = 10 pA).

Our investigation revealed a noteworthy capability of our methodology to induce reversible transformations in topological pentacene polymer segments. As illustrated in Figure 5, we successfully achieved the ON/OFF switching of topological states at the termini of both the right and left segments through the sudden addition of a single H atom to the pentacene backbone. While occurrences of this phenomenon have been observed during various dehydrogenation processes, specific parameters governing the addition of the single H atom have not yet been identified. However, we propose a plausible approach involving tip-induced hydrogen migration from a nearby polymer. Figure 5 demonstrates the reversibility of this process, indicating that the OFF state (Figure 5b,d) may occur even in cases in which hybridized states have already formed (Figure 5a,c). This phenomenon is observed as a stark decreases in conductance only at the edge of the nontrivial segment containing the hydrogen pair, while the opposite edge state remains intact (see Figures 5 and S10).

As described above, the addition of a hydrogen pair to an aromatic-pentacene molecule increases the number of Clar sextets from one to two, thus enhancing its chemical stability. For a quinoid-pentacene unit, the addition of two hydrogen pairs to the carbon unit does not modify the number of sextets (they keep two). On the other hand, the addition of the two extra hydrogens into the same aromatic ring represents an abrupt break of the aromatic conjugation of the pentacene unit leading to a π-electron arrangement like that on anthracene aromaticity. In this case, the double-hydrogenated pentacene behaves like an anthracene monomer, thus not showing topological phases. In fact, as commented above, the bandgap of double-hydrogenated pentacene polymer is very similar to those measured in anthracene polymers on Au(111). When a single H atom is absorbed onto a quinoid-pentacene unit, the situation becomes particularly intriguing. This addition induces the emergence of an additional sp^2^ radical, maintaining the total number of sextets at two (see Figure S11). However, as expected from Lieb’s theorem, our experimental data do not reveal any evidence of radical character surrounding the singly hydrogenated pentacene unit, as it feasibly recombines into a closed-shell configuration with one of the existing edge state radicals as has been reported in graphene structures,^54^ graphene nanoribbons,^40,42^ nanographenes,^55,56^ and even pentacene oligomers.^32^

## Conclusions

In summary, by leveraging a combination of low-temperature STM/AFM imaging and spectroscopy, we successfully demonstrated the controlled manipulation of the coupling between topological edge states in precisely engineered heterostructures of pentacene polymers. The key parameter controlling the topological class of a pentacene polymer segment is its extension, which can be finely tuned by selectively adding hydrogen atoms to specific pentacene units with single-atom precision. We found that altering the extensions of the semiconducting barrier between adjacent topological states enables the reversible creation of customizable topological heterostructures with tunable electronic and magnetic properties. Notably, we observed that the edge states couple in an antiferromagnetic fashion, with the state extending over a few nanometers. This configuration holds promise for integrating pentacene polymer components into future nanoelectronics. Our findings advance not only the fundamental understanding of topological phases in carbon molecular nanostructures but also the development of quantum devices and highly conductive long molecular wires featuring a narrow bandgap.

## Methods

### SPM Experiments

Experiments were carried out within an ultrahigh vacuum (UHV) system with a pressure below 5 × 10^–10^ mbar, using a commercial scanning tunneling microscope (STM) and noncontact atomic force microscope (nc-AFM) from CreaTec Fischer & Co. GmbH. The experiments were conducted at low temperature (4.2 K). The images were acquired by using a Pt/Ir tip attached to a qPlus sensor (resonant frequency ≈30 kHz; stiffness ≈1800 N m^–1^), while a bias voltage is applied to the sample. For nc-AFM images, qPlus sensor was operating at frequency modulation mode with an oscillation amplitude of 50 pm. Sharp metallic tips were achieved through gentle indentations in the bare surface sample, and then, a single CO molecule was picked up by the tip previously dosed on the cold sample (*T* < 10 K). The Au(111) sample was cleaned by Ar^+^ sputtering at 1 keV and then annealing at 800 K.

Conductance d*I/*d*V* spectra and maps were acquired with a conventional lock-in technique with modulations of 5 and 10 mV, respectively. For the Kondo resonance spectroscopies versus temperature, the sample were heated from 4.2 K by using a Zener diode. Then, the spectra were fitted by using the Frota function,^57^ and the Kondo temperature is extracted from the Fermi liquid model:  with an empirical parameter of α = 4.48 ± 0.12. For the analysis of the states hybridization, the perturbative model of the Markus Ternes program^46^ was used in order to fit the experimental spectroscopies and obtaining the magnetic exchange coupling *J*_eff_. The images were analyzed in WSxM software.^58^

The molecular precursor 4BrPn (6,13-bis(dibromomethylene)-6,13-dihydropentacene) was outgassed in UHV for several hours and then thermally sublimated onto the clean Au(111) surface kept at UHV and room temperature conditions from a tantalum crucible maintained at 200 °C. After an annealing step at 320 °C for the formation of pentacene polymers, the sample was transferred to the STM stage held at 4.2 K for the characterization.

### Hydrogenation and Dehydrogenation Process

After the formation of the pentacene polymers, molecular hydrogen was inserted into the UHV chamber with a pressure of 5 × 10^–10^ mbar and the sample was dosed for 10 min. Meanwhile, the ion gauge was on to induce the break of H_2_ in atomic hydrogen. Then, the sample was inserted into the SPM head.

For the dehydrogenation method, the tip is positioned on top of a pentacene monomer, and then, the bias voltage is varied from −1.5 to −2.6 V with a constant current of 10 pA. Usually, a change in the tip–sample distance is visible at −2.5 V (see Figure S1). For the hydrogen cleaning in a section of the polymer, a bias voltage of −2.5 V was applied, while the tip is scanning through the desired region with a relatively slow time/line speed (≈3 s/line).

### Theoretical Calculations

We employed the Huckel model with distance-dependent hoppings and many-body calculations for the Hubbard model solved by means of the CASCI (Complete Active Space–Configuration Interaction) method. We take reference values for the hoppings and electrostatic repulsion on *p*_*z*_ orbitals to be, respectively, *t* = −2.8 eV and *U* = 4.3 eV.^59^ This hopping corresponds to a standard average bond length of 1.40 Å in aromatic compounds. The central hydrogenated units are modeled by anthracene units (see Figures S6 and S7).

From the many-body CAS calculation, we can estimate the spectral gap between the singlet ground state and the first excited triplet state, which corresponds to the experimentally observed exchange coupling between zero-bias resonance states. We depart from the Hubbard Hamiltonian with the distance-dependent hoppings:
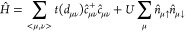
1where the parametrization is given by
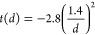
2

In such model, we can reproduce the nontrivial end states by setting up the bond lengths on the pentacene bridges, *d*_p_, to be 1.55 Å. The bond lengths in the bridges between anthracene units, *d*_a_, are set to 0.8 Å to reproduce the electronic gap in the Huckel Hamiltonian (see Figure S7). The difference between the trivial and nontrivial phase is represented in Figure S6.

To estimate the spectral gap, we resort to the CAS(4,4) method, in which we first diagonalize the one-electron part and select the four main molecular orbitals around the Fermi energy (see Figure S8). We can then change basis from the atomic sites to the molecular orbitals
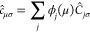
3where the greek subscripts label atomic sites; latin subscripts label molecular orbitals (the one-electron eigenstates obtained from (1) by setting *U* = 0); small letters, *c*, are used for the creation/annihilation operators on atomic sites; and large letters, *C*, for the creation/annihilation operators on molecular orbitals. The ϕ_μ_(*j*) is the coefficient on site μ of the expansion of the *j*-th molecular orbital on the basis of atomic sites.

We introduce (3) in the original Hamiltonian (1) and keep only the creation/annihilation operators of the selected active space, which in this case is formed by the four orbitals around the Fermi level (HOMO–1, HOMO, LUMO, LUMO + 1). The resulting Hamiltonian can be exactly diagonalized numerically.

The ground state is always degenerated because of the presence of noncommunicating end states on both pentacene chains. We then take the difference between the third and first eigenvalues of this Hamiltonian, which yields the exchange coupling, and which we can then calculate for each number of hydrogenated units (modeled as anthracenes in the Hubbard calculation). As shown in Figure S9, this coupling rapidly decays to zero, resulting in degenerate ground states due to the noncommunicating radicals.

## Supporting Information Available

Details of the methods used in this experiment (SPM parameters, preparation of the sample, hydrogenation and tip-induced dehydrogenation process, proposed theoretical calculations and parameters); additional SPM images of tip-induced dehydrogenation, characterization of electronic structure, and formation of topological heterostructures in pentacene polymers; theoretical graphics for the comparison with experiments and chemical model explanation for the reversible hydrogenation case; and tables of magnetic exchange couplings for different cases of the hybridized topological edge states (PDF)
