# Supplementary material for: Atomically Precise Control of Topological State Hybridization in Conjugated Polymers
Source: ACS Nano. 2024 Oct 15;18(43):29902–12. doi: 10.1021/acsnano.4c10357 (PMC11526428; doi:10.1021/acsnano.4c10357)
Supplement: Supplementary file 1 — nn4c10357_si_001.pdf [file nn4c10357_si_001.pdf]

SUPPLEMENTARY INFORMATION

# Atomically Precise Control of Topological State Hybridization in Conjugated Polymers

*Alejandro Jiménez-Martín,[1,2,3] Zdenka Sosnová,[2] Diego Soler,[2] Benjamin Mallada,[1,2] Héctor González-Herrero,[1,4,5] Shayan Edalatmanesh,[1,2] Nazario Martín,[6,7] David Écija,[7] Pavel Jelínek,[1,2]* and Bruno de la Torre[1,8]**

[1] Regional Centre of Advanced Technologies and Materials, Czech Advanced Technology and Research Institute (CATRIN), Palacký University, 78371 Olomouc, Czech Republic.

[2] Institute of Physics of the Czech Academy of Sciences, 16200 Prague, Czech Republic.

[3] Faculty of Nuclear Sciences and Physical Engineering, Czech Technical University, 11519 Prague, Czech Republic.

[4] Departamento de Física de la Materia Condensada, Universidad Autónoma, E-28049 Madrid, Spain.

[5] Condensed Matter Physics Center (IFIMAC), Universidad Autónoma, E-28049 Madrid, Spain.

[6] Departamento de Química Orgánica, Facultad de Ciencias Químicas, Universidad Complutense, 28040 Madrid, Spain.

[7] IMDEA Nanoscience, Campus Universitario de Cantoblanco, 28049 Madrid, Spain.

[8] Nanomaterials and Nanotechnology Research Center (CINN), CSIC-UNIOVI-PA, 33940 El Entrego, Spain.

* Correspondence to: jelinekp@fzu.cz (P.J.) and b.delatorre@cinn.es (B.T.)

**Contents**

Methods
Figures S1 to S11
Table 1 and 2
References

## Methods

SPM experiments

Experiments were carried out within an ultra-high vacuum (UHV) system with a pressure below 5 x $10^{-10}$ mbar, using a commercial scanning tunneling microscope (STM) and non-contact atomic force microscope (nc-AFM) from CreaTec Fischer & Co. GmbH. The experiments were conducted at low temperature (4.2 K). The images were acquired by using a Pt/Ir tip attached to a qPlus sensor (resonant frequency ≈ 30 kHz; stiffness ≈ 1800 N $m^{-1}$) while a bias voltage is applied to the sample. For nc-AFM images, qPlus sensor was operating at frequency modulation mode with an oscillation amplitude of 50 pm. Sharp metallic tips were achieved through gentle indentations in the bare surface sample and then, a single CO molecule was picked up by the tip previously dosed on the cold sample (T < 10 K). The Au(111) sample was cleaned by cycles of $Ar^+$ sputtering at 1 keV followed by annealing at 800 K.

Conductance dI/dV spectra and maps were acquired with a conventional lock-in technique with a modulation of 5 and 10 mV, respectively. The Kondo resonance spectroscopies versus temperature the sample was heated from 4.2 K by using a Zener diode. Then, the spectra were fitted by using the Frota function[1] and the Kondo temperature is extracted from the Fermi-liquid model: $\Gamma = \frac{1}{2}\sqrt{(\alpha K_B T)^2 + (2K_B T_K)^2}$ with an empirical parameter of α = 4.48 ± 0.12. For the analysis of the states hybridization, the perturbative model of the Markus Ternes program[2] was used in order to fit the experimental spectroscopies and obtaining the magnetic exchange coupling $J_{eff}$. The images were analyzed in WSxM software.[3]

The molecular precursor 4BrPn (6,13-bis(dibromomethylene)-6,13-dihydropentacene) was outgassed in UHV for several hours and then thermally sublimated onto the clean Au(111) surface kept at UHV and room temperature conditions from a tantalum crucible maintained at 200 ºC. After annealing step at 320 ºC for the formation of pentacene polymers, the sample was transferred to the STM stage held at 4.2 K for the characterization.

Hydrogenation and dehydrogenation process

After the formation of the pentacene polymers, molecular hydrogen was inserted into the UHV chamber with a pressure of 5 x $10^{-10}$ mbar and the sample was dosed for 10 minutes. Meanwhile, the ion gauge was on to induce the break of the $H_2$ in atomic hydrogen. Then the sample was inserted into the SPM head.

For the dehydrogenation method, the tip is positioned on top of a pentacene monomer and then the bias voltage is variated from -1.5 to -2.6 V with a constant current of 10 pA. Usually, a change in the tip-sample distance is visible at -2.5 V, see Figure S1. For the hydrogen cleaning in a section of the polymer, a bias voltage of -2.5V was applied while the tip is scanning through the desired region with a relative slow time/line speed (≈ 3s/line).

Theoretical calculations

We have employed the Huckel model with distance-dependent hoppings and many-body calculations for the Hubbard model solved by means of the CASCI (Complete Active Space – Configuration Interaction) method. We take reference values for the hoppings and electrostatic repulsion on $p_z$ orbitals to be, respectively, t = -2.8 eV and U = 4.3 eV.[4] This hopping corresponds to a standard average bond length of d = 1.40 Å in aromatic compounds. The central hydrogenated units are modeled by anthracene units (see Figure S6 and S7).

From the many-body CAS calculation, we can estimate the spectral gap between the singlet ground state and the first excited triplet state, which corresponds to the experimentally observed exchange coupling between zero-bias resonance states. We depart from the Hubbard Hamiltonian with the mentioned distance-dependent hoppings:

$$\hat{H} = \sum_{<\mu,\nu>} t(d_{\mu\nu})\, \hat{c}^{+}_{\mu\nu}\, \hat{c}_{\mu\nu} + U \sum_{\mu} \hat{n}_{\mu\uparrow}\hat{n}_{\mu\downarrow} \qquad (1)$$

Where the parametrization is given by:

$$t(d) = -2.8\left(\frac{1.4}{d}\right)^{2} \qquad (2)$$

In such model, we can reproduce the non-trivial end-states by setting up the bond-lengths on the pentacene bridges, $d_p$, to be 1.55Å. The bond lengths in the bridges between anthracene units, $d_a$, are set to 0.8 Å to reproduce the electronic gap in the Huckel Hamiltonian (see Figure S7). The difference between the trivial and non-trivial phase is represented in Figure S6.

To estimate the spectral gap, we resort to the CAS(4,4) method, in which we first diagonalize the one-electron part and select the four main molecular orbitals around Fermi energy (see Figure S8). We can then change basis from the atomic sites to the molecular orbitals

$$\hat{c}_{\mu\sigma} = \sum_{j} \phi_j(\mu)\, \hat{C}_{j\sigma} \qquad (3)$$

Where the greek subscripts label atomic sites, latin subscripts label molecular orbitals (the one-electron eigenstates obtained from (1) by setting U=0), small letters, c, are used for the creation/annihilation operators on atomic sites and large letters, C, for the creation/annihilation operators on molecular orbitals. The $\phi_{\mu}(j)$ is the coefficient on site $\mu$ of the expansion of the j-th molecular orbital on the basis of atomic sites.

We introduce (3) in the original Hamiltonian (1) and keep only the creation/annihilation operators of the selected active space, which in this case is formed by the four orbitals around Fermi level (HOMO-1, HOMO, LUMO, LUMO+1). The resulting Hamiltonian can be exactly diagonalized numerically.

The ground state is always degenerate because of the presence of non-communicating end states on both pentacene chains. We then take the difference between the third and first eigenvalues of this Hamiltonian, which yields the exchange coupling and which we can then calculate for each number of hydrogenated units (modeled as anthracenes in the Hubbard calculation). As shown in Figure S9, this coupling rapidly decays to zero, resulting in degenerate ground-states due to the non-communicating radicals.

**Figures S1 to S11**

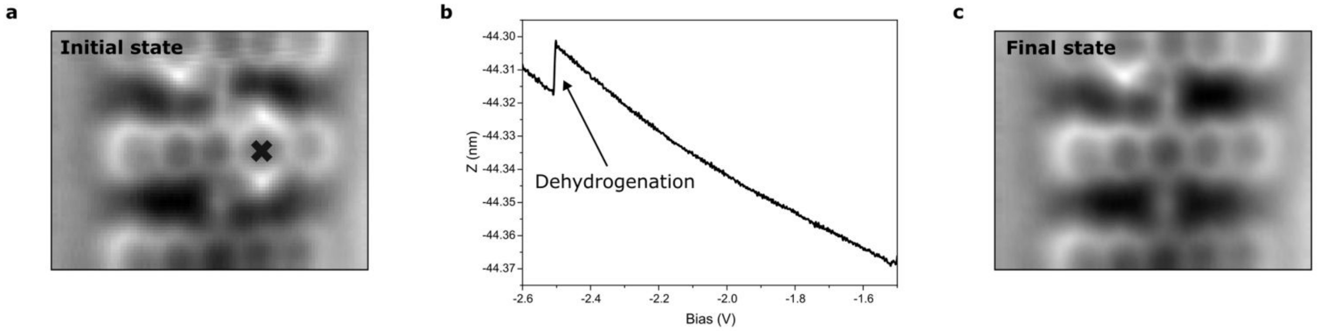


**Figure S1. Dehydrogenation process in a pentacene monomer.** a) detailed AFM image of the initial state with a double-hydrogenated pentacene monomer. b) STM tip height spectroscopy of the dehydrogenation ramp showing the instant when the hydrogen is removed. c) close-up AFM image of the pentacene monomer final state after dehydrogenation.

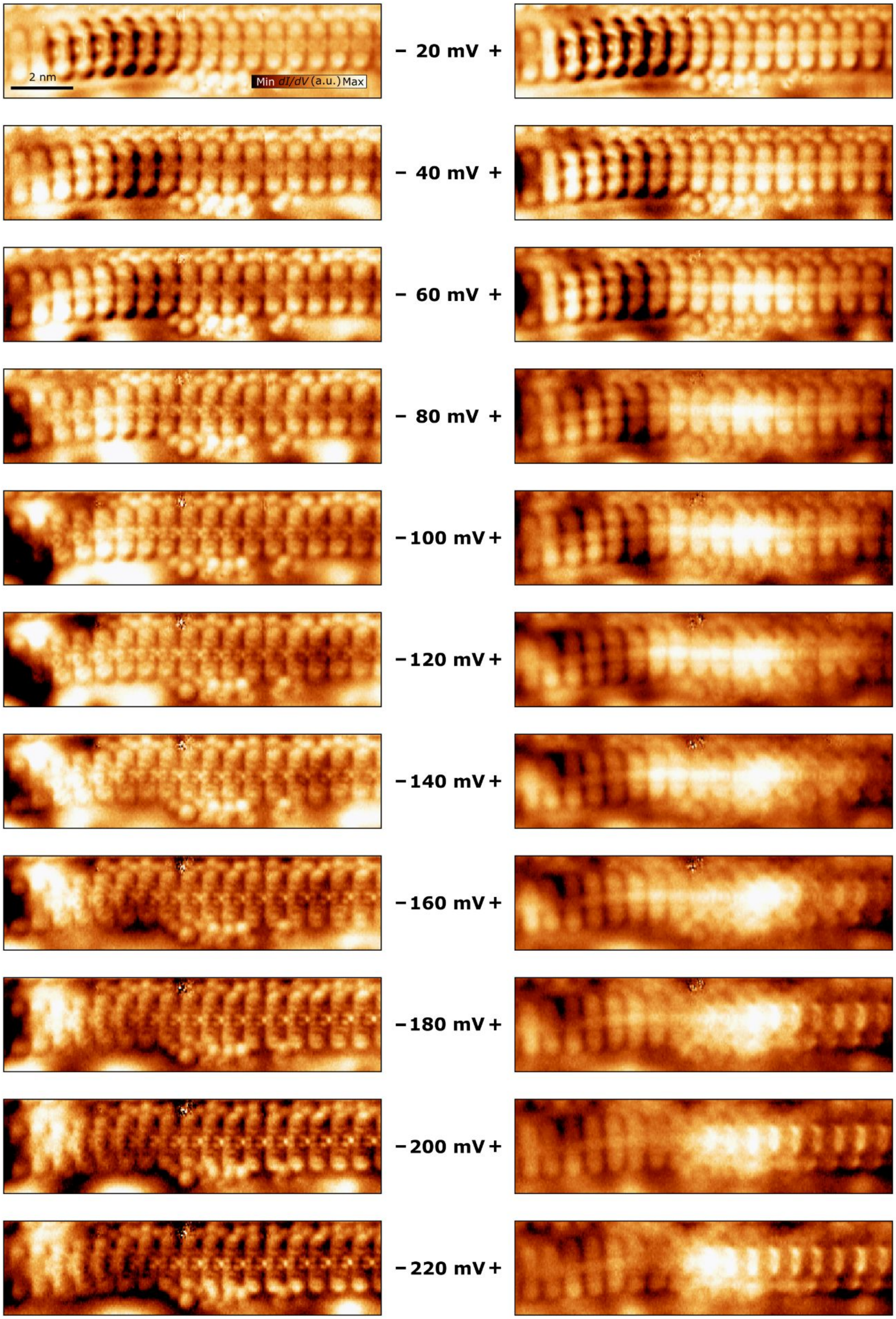


**Figure S2. Constant-current conductance maps of the topological edge state.** *dI/dV* maps at the edge of the pristine pentacene polymer by steps of 20 mV at positive (left column) and negative (right column) values. (100 pA)

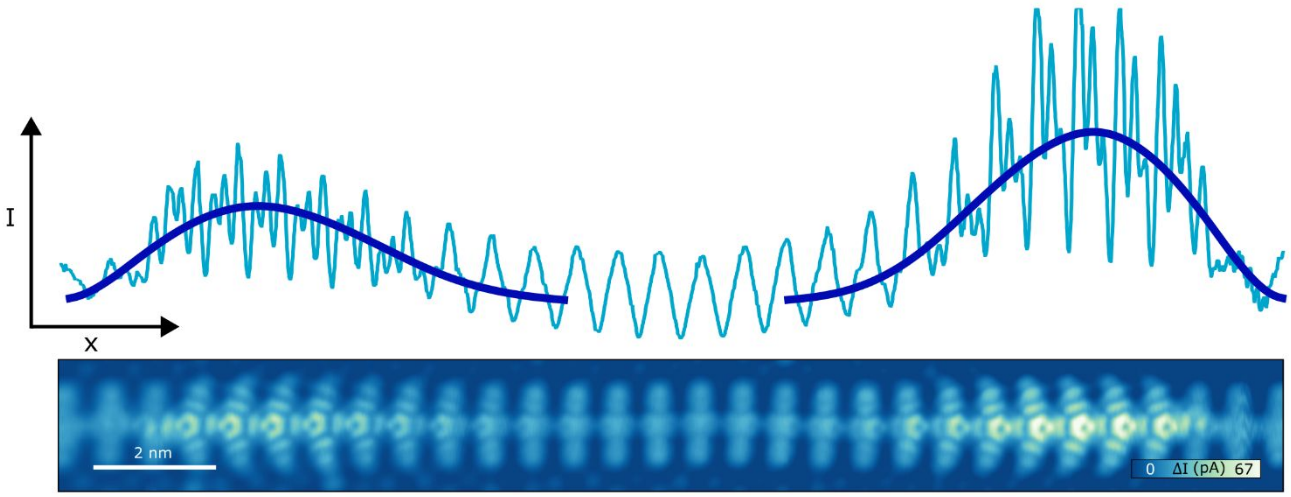


**Figure S3. Kondo resonance distribution in the pentacene polymer.** The constant height STM image shows a non-trivial phase with the Kondo resonance features at the edge of the region and the corresponding profile of the current along the pentacene polymer. (5 mV; 10 pA)

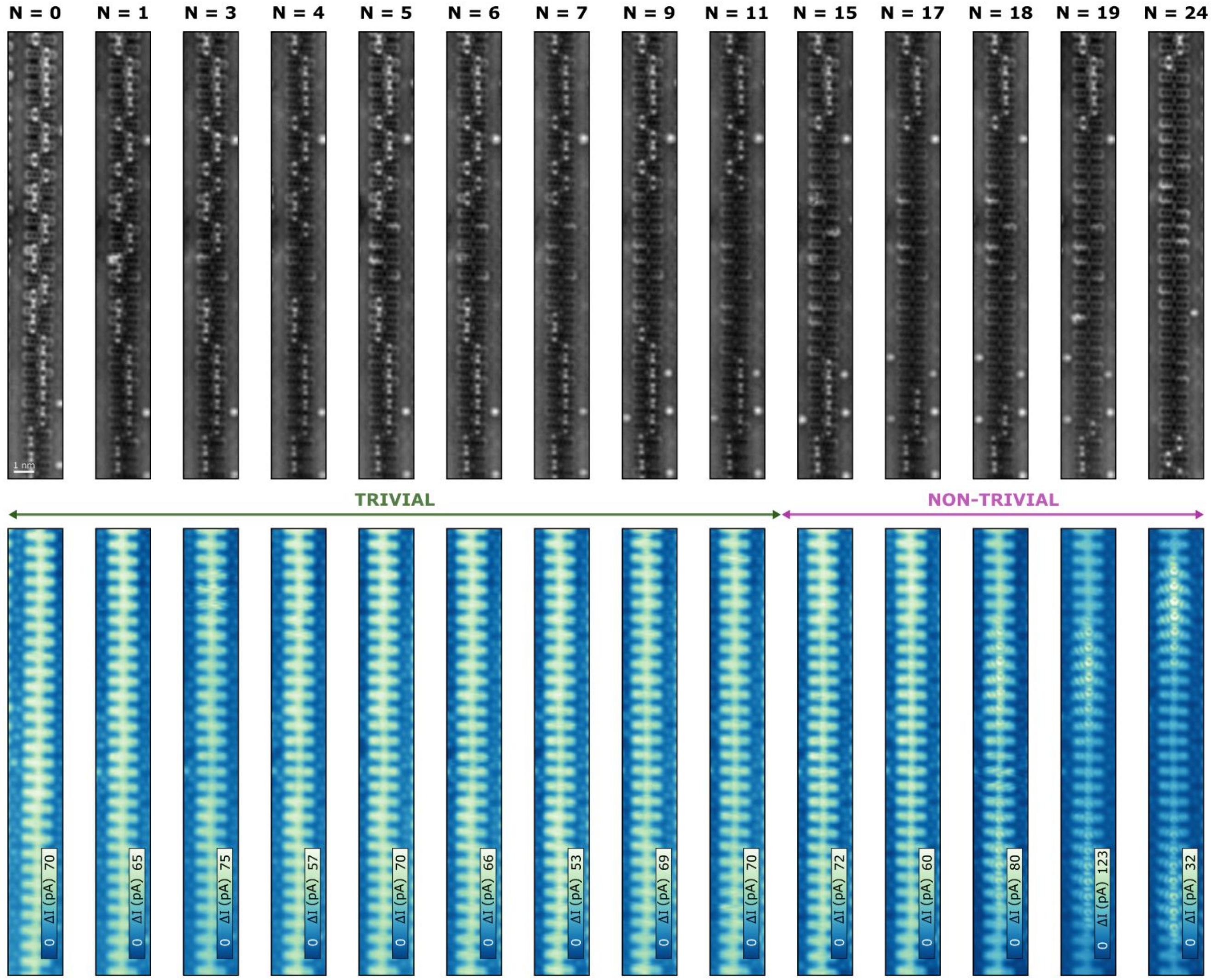


**Figure S4. Creation of non-trivial region in a single pentacene polymer.** nc-AFM (up) and STM (down) images of the dehydrogenation process for each N case in the formation of the non-trivial phase. (5 mV; 10 pA)

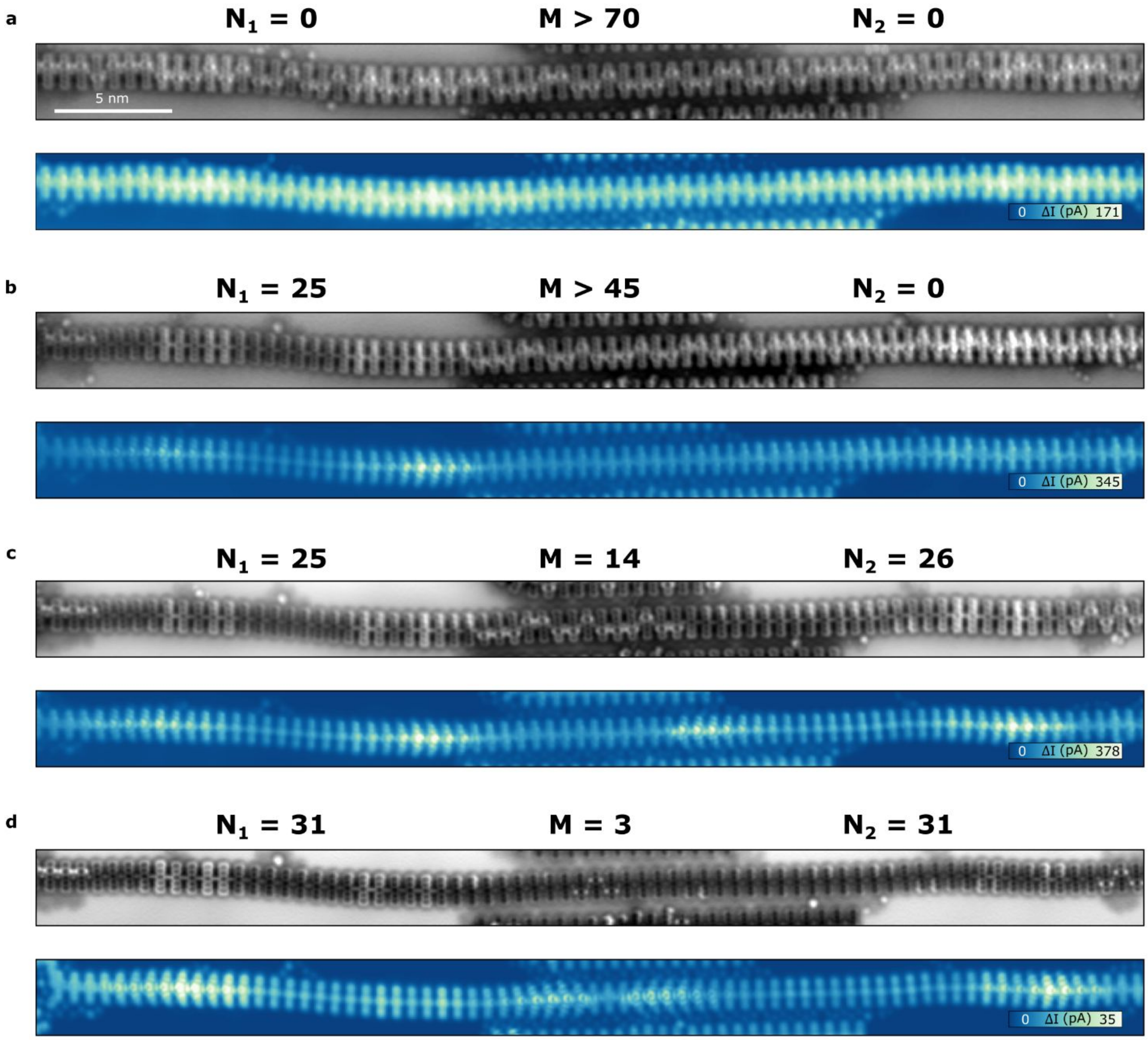


**Figure S5. Creation of heterostructure in a single pentacene polymer.** nc-AFM and STM images of a) Hydrogenated pentacene polymer, b) N = 25 dehydrogenated pentacene units, c) M = 14 hydrogenated pentacene monomers forming the first semiconducting barrier, d) Final state of the heterostructure with M = 3 and $N_{1,2} > 17$. (5 mV; 10 pA)

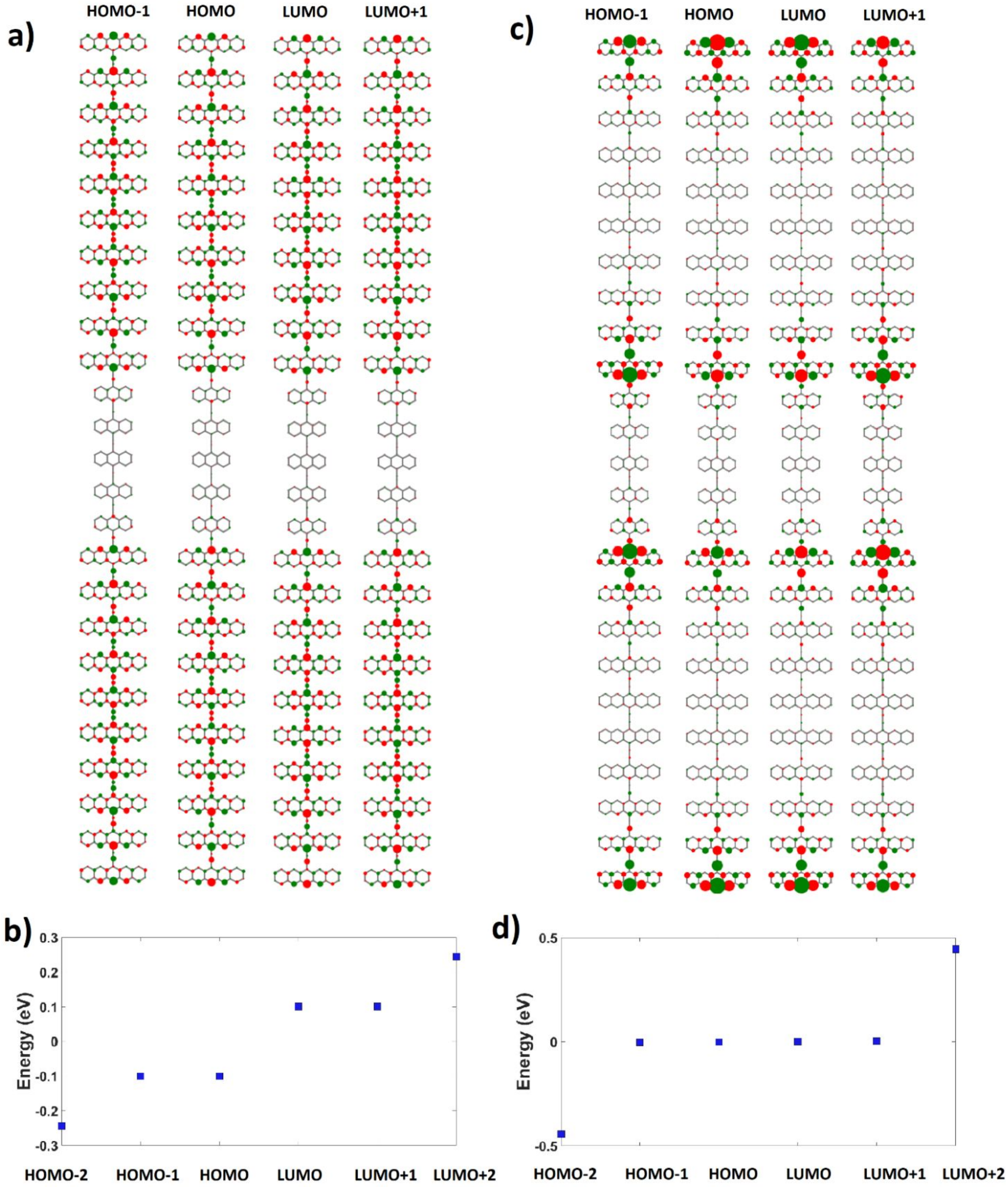


**Figure S6. Topological phase transition in the Huckel model.** On the left, one-electron Huckel orbitals **a)** and corresponding energy spectrum **b)**, for the topologically trivial phase, where the distance $d_p$ in the pentacene bridges is set to 1.15 Å eV. On the right, one-electron Huckel orbitals **c)** and corresponding energy spectrum **d)**, for the non-trivial phase, with distance $d_p$ in the pentacene bridges set to 1.55 Å eV. In both cases, the distance $d_a$ on the bridges between anthracene units is set to 0.8 Å. The orbitals and the spectra are obtained from diagonalizing the Huckel Hamiltonian (first-neighbours tight-binding) where all distances are set to 1.40 Å (corresponding to a hopping of t=-2.8 eV) except the ones on the bridges.

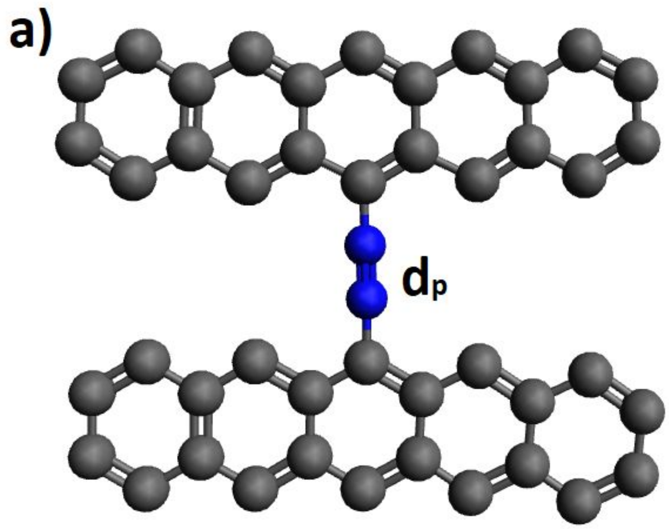


**Figure S7. Graphical scheme of the distance $d_p$ and $d_a$ between the carbons of the bridges of, respectively, pentacene and anthracene units.** All other non-highlighted distances are set to 1.40 Å.

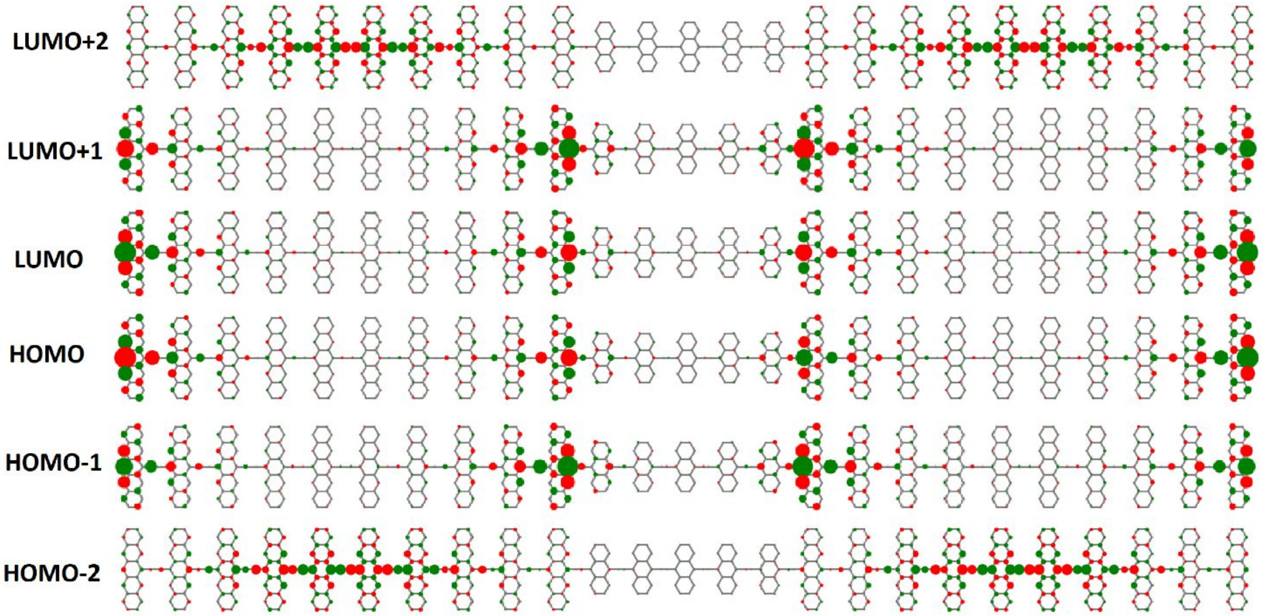


**Figure S8. Molecular orbitals included in the active space of CASCI calculations.** Six molecular orbitals obtained from one-electron Huckel calculation employed in the CASSCI(6,6) calculation.

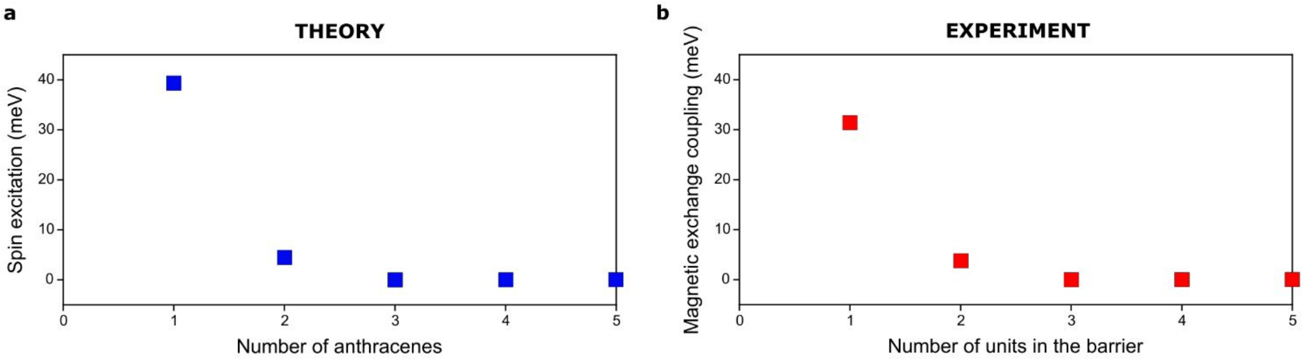


**Figure S9. Dependence of the spin excitation with the barrier in heterostructures.** a) Spin excitation calculated from CAS(6,6) for the Hubbard model. Results are identical for CAS(4,4) and CAS(8,8), showing that the CASCI calculations are converged. The spin excitation is calculated from the difference between the singlet ground state and the excited triplet state of the Hubbard model. b) Experimental values of the magnetic exchange interaction $J$ for different hydrogenated pentacene units in the barrier between ZBRs.

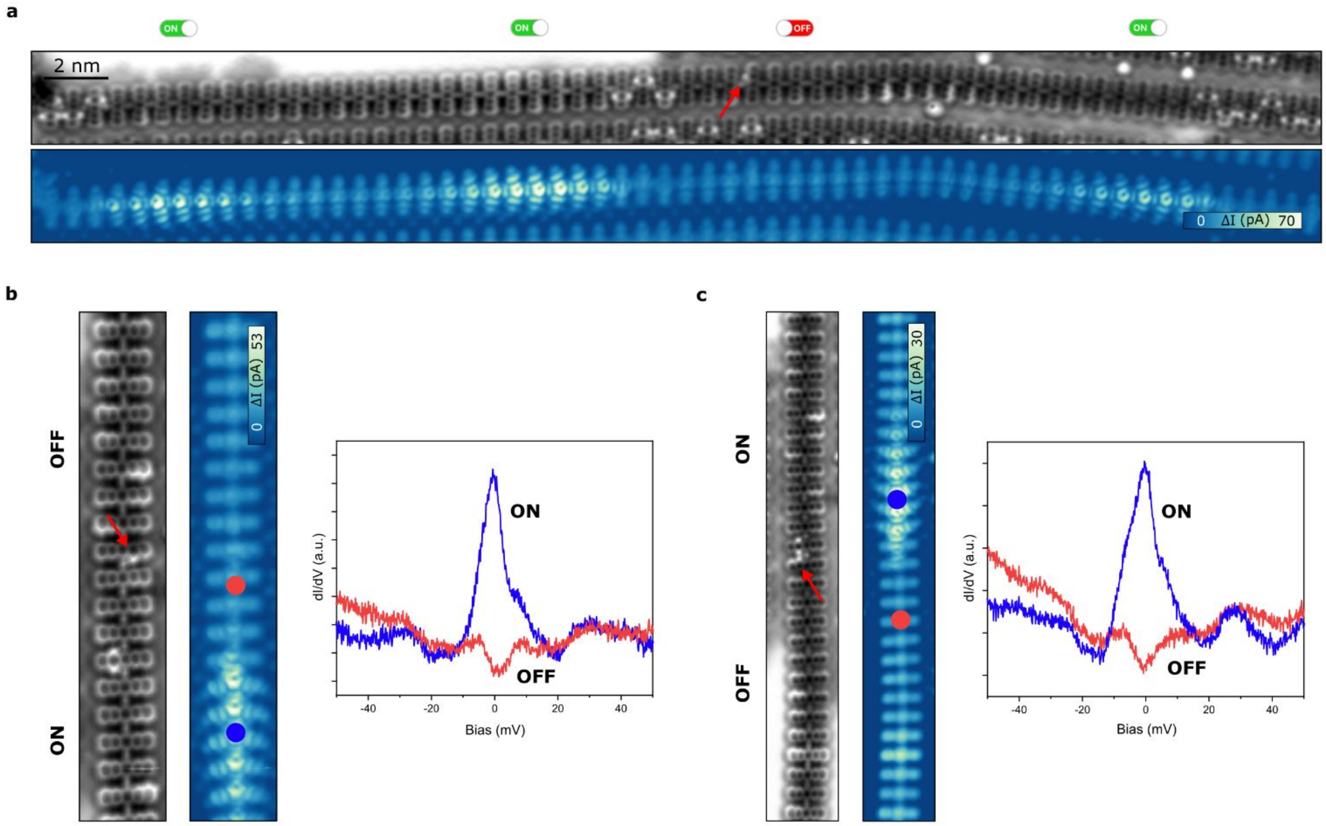


**Figure S10. Characterization of the extra H.** a) AFM and STM overview images of a complete heterostructure with the effect of an additional hydrogen in one of the non-trivial regions. AFM, STM and STS characterization of the a) up and b) down extra hydrogenation and his effect in the state when M = 1. (5 mV; 10 pA)

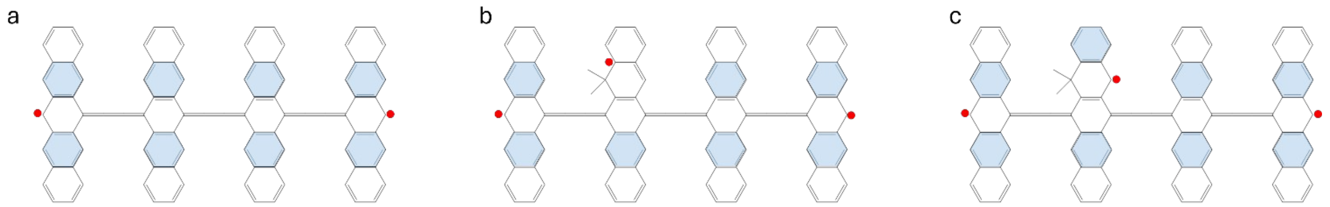


**Figure S11. Effect of a single additional hydrogen atom to a carbon unit in a quinoidal pentacene polymer.** a) pristine quinoidal pentacene polymer (non-trivial topology). b) and c) an additional hydrogen atom is attached to a carbon leading to the formation of a single Clar sextet b) or, more favourably, two Clar sextets c) in the pentacene unit.

**Table 1 and 2**

| J (meV) | N = 15 | N = 16 | N = 17 | N = 18 |
|---|---|---|---|---|
| Polymer 11 | 3.96 | 2.36 | - | - |
| Polymer 13 | - | - | 5.53 | 3.06 |

**Table 1. Exchanges values (J) for the intrapolymer case.** The values of the magnetic exchange for two different polymers are displayed when the non-trivial region is created.

| J (meV) | M = 2 | M = 1 |
|---|---|---|
| Polymer 1 | 3.85 | 32.42 |
| Polymer 5 | 3.39 | 31.63 |
| Polymer 7 | 3.87 | - |
| Polymer 10 | - | 31.45 |

**Table 2. Exchanges values (J) for the interpolymer case.** The table shows different magnetic couplings according to the region size in different polymers.

## References


(1) Frota, H. O. Shape of the Kondo Resonance. *Phys. Rev. B* **1992**, *45* (3), 1096–1099.

(2) Ternes, M. Spin Excitations and Correlations in Scanning Tunneling Spectroscopy. *New J. Phys.* **2015**, *17* (6), 063016.

(3) Horcas, I.; Fernández, R.; Gómez-Rodríguez, J. M.; Colchero, J.; Gómez-Herrero, J.; Baro, A. M. WSXM: A Software for Scanning Probe Microscopy and a Tool for Nanotechnology. *Review of Scientific Instruments* **2007**, *78* (1), 013705.

(4) Schüler, M.; Rösner, M.; Wehling, T. O.; Lichtenstein, A. I.; Katsnelson, M. I. Optimal Hubbard Models for Materials with Nonlocal Coulomb Interactions: Graphene, Silicene, and Benzene. *Phys. Rev. Lett.* **2013**, *111* (3), 036601.
